# Editorial: Drug Repositioning: Current Advances and Future Perspectives

**DOI:** 10.3389/fphar.2018.01068

**Published:** 2018-09-20

**Authors:** Yuhei Nishimura, Hideaki Hara

**Affiliations:** ^1^Department of Integrative Pharmacology, Mie University Graduate School of Medicine, Tsu, Japan; ^2^Molecular Pharmacology, Department of Biofunctional Evaluation, Gifu Pharmaceutical University, Gifu, Japan

**Keywords:** computational drug repositioning, integrative strategies, clinical database, data sharing, patenting

Drug repositioning (DR) is the process of identifying new indications for existing drugs. At present, the conventional *de novo* drug discovery process requires an average of about 14 years and US$2.5 billion to approve and launch a drug (Nosengo, 2016). DR can reduce the time and cost of this process because it takes advantage of drugs already in clinical use for other indications or drugs that have cleared phase I safety trials but have failed to show efficacy for the intended diseases. Historically, DR has been realized through serendipitous clinical observations or improved understanding of disease mechanisms. However, recent technological advances have enabled more systematic approaches to DR.

It has been widely recognized that most small-molecule drugs interact with more than one target protein (Paolini et al., 2006; Mestres et al., 2008). Understanding of the polypharmacology is a crucial aspect of DR (Lavecchia and Cerchia, 2016). Various *in silico* methods have been developed to apply the polyphramacology for DR, including omics based (Nagaraj et al., 2018) and molecular docking based (Xu et al., 2018) approaches. March-Vila et al. and Tan et al. present overviews about the computational methods for DR (March-Vila et al.; Tan et al.). Various *in vitro* assays have been performed to systematically assess the biological function of drugs. These drugs' bioactivities, combined with their chemical structure, physical properties, and clinical indications, have been recorded in various public databases, such as PubChem (Kim et al., 2016), CheEMBL (Gaulton et al., 2017), DrugBank (Wishart et al., 2018), and DrugCentral (Ursu et al., 2017). The concept that similar drugs (in terms of their functions and/or structures) may have similar clinical indications has been widely used in DR. If drug A has bioactivities similar to those of drug B, which has been approved to treat disease X, it is plausible that drug A may also treat disease X. Transcriptional responses induced by drugs and diseases can also be used in DR. If the transcriptional signature of drug C is inversely correlated to that of disease Y and/or positively correlated to that of drug D, which has been used to treat disease Y, it is likely that drug C may be used to treat disease Y. Representative resources for this approach are the Connectivity Map (Lamb et al., 2006) and the Library of Integrated Network-based Cellular Signatures (LINCS; Subramanian et al., 2017; Keenan et al., 2018; Koleti et al., 2018). Additionally, similarity of protein structures, especially for the ligand binding site, can be useful information in DR. If protein A, a key molecule of disease Z (for which no therapeutics exist), has a local structure similar to that of protein B, which is known as a therapeutic target of drug E, one can predict that drug E may be used to treat disease Z. Various databases are useful for this approach, including Protein Data Bank (PDB; Rose et al., 2017), Protein Binding Sites (ProBis; Konc and Janezic, 2014), and Protein-Ligand Interaction Profiler (PLIP; Salentin et al., 2015). Integrating these approaches can extend the domain of applicability of each method and provide novel information. Representative databases for these integrative approaches are the Drug Repurposing Hub (Corsello et al., 2017), Drug Target Commons (Tang et al., 2018), and Open Targets (Koscielny et al., 2017). Databases that have information about clinical results of DR have also been developed, including repoDB (Brown and Patel, 2017) and repurposeDB (Shameer et al., 2018).

Combining *in silico* prediction and *in vitro* validation, Hamdoun et al. found that anthelmintic niclosamide can be used to treat multidrug-resistant leukemia (Hamdoun et al.). Fang et al. developed an integrated systems pharmacology approach for DR of natural produce targeting aging-associated disorders (Fang et al.). DR of natural products have also been reported, including ginkgolide C for myocardial ischemia/reperfusion-induced inflammatory injury (Zhang et al.), halofuginone for osteoarthritis (Mu et al.), nardosinone for alveolar bone resorption (Niu et al.), and pleuromutilins for infections due to *Staphylococcus aureus* (Dong et al.). Takai and Jin reviewed the possibility of chymase inhibitors as a novel therapeutic agent for non-alcoholic steatohepatitis (Takai and Jin).

Retrospective analysis of clinical records can be used to confirm the validity of DR. Proton pump inhibitors, H^+^/K^+^-ATPase inhibitors, have been reported to protect cisplatin-induced nephrotoxicity through inhibition of renal basolateral organic cation transporter 2 and to enhance the sensitivities of anticancer agents by inhibiting V-ATPase in tumor cells (Ikemura et al., 2017). These off-target effects of proton pump inhibitors have been successfully validated by retrospective analysis of electronic health records (Ikemura et al.; Wang et al., 2017). The inhibitory effects of statin for carcinogenesis in various tissues, including prostate, have been demonstrated in a number of experimental studies (Thurnher et al., 2012; Yu et al., 2014). Chen et al. demonstrated that simvastatin reduced the risk of prostate cancer mortality in patients with hyperlipidemia using a health insurance research database (Chen et al.). Sharing clinical records such as electronic health records, health insurance records, and clinical trial data, can be effective for determining DR.

High throughput screening of chemicals using *in vitro* and/or *in vivo* systems can also strongly drive DR (Nishimura and Hara, 2016). However, most *in vitro* systems currently used for high throughput screening are two-dimensional monolayer cultures that differ from physiological conditions. Langhans reviewed the three-dimensional *in vitro* cell culture models that may recapitulate microenvironmental factors that resemble *in vivo* tissue and disease pathology and discussed the significance and challenges of the system in DR (Langhans).

Low-dose metronomic chemotherapy has emerged as a regimen that can alter the tumor environment and suppress innate features supporting tumor growth by targeting not only tumor cells but also endothelial and immune cells (Loven et al., 2013). The concept of low-dose metronomic chemotherapy has been successfully used in DR (Hashimoto et al., 2010; Pasquier et al., 2011). Quirk and Ganapathy-Kanniappan hypothesized that current chemotherapeutics at sub-lethal, non-toxic doses might up-regulate MHC-class I chain related protein A or B and enhance the efficacy of immunotherapy mediated by natural killer cells that recognize these proteins (Quirk and Ganapathy-Kanniappan). Detailed investigation is necessary to further validate this hypothesis.

Patenting in DR can be challenging, especially if the novel indications have already been claimed by competitors within the same drug class (Sternitzke, 2014). Mucke provided useful strategies for patenting in DR, suggesting the importance of systematic collections of DR patent documents and the expert systems that assist researchers in extracting relevant patent information (Mucke).

The regulatory system for approval can also significantly affect the stream of DR. Nishimura et al. provided perspectives and future directions for DR, including an approval system suitable for DR (Nishimura et al.).

This research topic will maximize knowledge of DR, with the hope of identifying drugs that can be exploited to prevent and/or treat diseases for which effective medications are currently lacking.

## Author contributions

YN drafted the editorial. Both authors revised and approved it.

### Conflict of interest statement

The authors declare that the research was conducted in the absence of any commercial or financial relationships that could be construed as a potential conflict of interest.
